# Cardiac implantable electronic devices in heart failure: needs, challenges, and real-world implementation

**DOI:** 10.1007/s10741-026-10672-w

**Published:** 2026-09-01

**Authors:** David Žižek, Miha Mrak, Anja Zupan Mežnar, Maja Ivanovski, Tadej Žlahtič, Christopher F. Barrientos, Andrew P. Ambrosy, Zlatko Fras, Mauro Biffi, Mitja Lainscak

**Affiliations:** 1 Department of Cardiology, University Medical Centre Ljubljana, Ljubljana, Slovenia; 2 Faculty of Medicine, University of Ljubljana, Ljubljana, Slovenia; 3 Kaiser Permanente San Francisco Medical Center, San Francisco, CA USA; 4 Department of Vascular Diseases, Division of Medicine, University Medical Centre Ljubljana, Ljubljana, Slovenia; 5 Institute of Cardiology, Cardiology Unit, IRCCS Azienda Ospedaliero Universitaria Di Bologna, Bologna, Italy; 6 General Hospital Murska Sobota, Murska Sobota, Slovenia

**Keywords:** Cardiac implantable electronic devices, Biventricular pacing, Conduction system pacing, Leadless pacing, Implantable cardioverter-defibrillator, Heart failure

## Abstract

Cardiac implantable electronic devices (CIEDs) are integral to heart failure (HF) management, providing therapies for bradyarrhythmias, cardiac resynchronization therapy (CRT), sudden cardiac death (SCD) prevention, and remote monitoring. This review summarizes current and potential future indications, emerging technologies, and challenges in the implementation of CIEDs in HF. Conventional right ventricular pacing in HF patients is increasingly being replaced by biventricular pacing (BVP) and left bundle branch area pacing (LBBAP), while leadless pacing pacemakers have emerged as an alternative to conventional transvenous systems, offering the potential to reduce lead- and device-related complications. As BVP remains the standard approach for CRT, recent data bestow LBBAP as a promising alternative and integration platform. Newer implantable cardioverter-defibrillator technologies, including subcutaneous and extravascular systems, may reduce lead-related complications while maintaining effective arrhythmia termination and recognition. Advances in atrial fibrillation management, implantable hemodynamic monitoring, and novel pacing indications such as atrioventricular recoupling and personalized pacing in HF with preserved ejection fraction are expanding the therapeutic potential of CIEDs. However, the adoption of these innovations is challenged by limited randomized evidence, procedural complexity, and substantial disparities in access and implementation across healthcare systems. Ongoing clinical trials will help define the role of emerging device technologies and monitoring strategies, while efforts to improve guideline adherence, value-based procurement, and equitable access remain essential to maximize the benefits of CIED therapy in patients with HF.

**Graphical abstract:**

HF – heart failure; LVEF – left ventricular ejection fraction; BVP – biventricular pacing; CSP – conduction system pacing; LP – leadless pacing; LBBAP – left bundle branch area pacing; RCT – randomized clinical trial; HFrEF – heart failure with reduced ejection fraction; CRT – cardiac resynchronization therapy; LOT-CRT – left optimized resynchronization therapy; AF – atrial fibrillation; TV-ICD – transvenous implantable cardiac defibrillator; SCD – sudden cardiac death; HFpEF – heart failure with preserved ejection fraction

**Figure:**
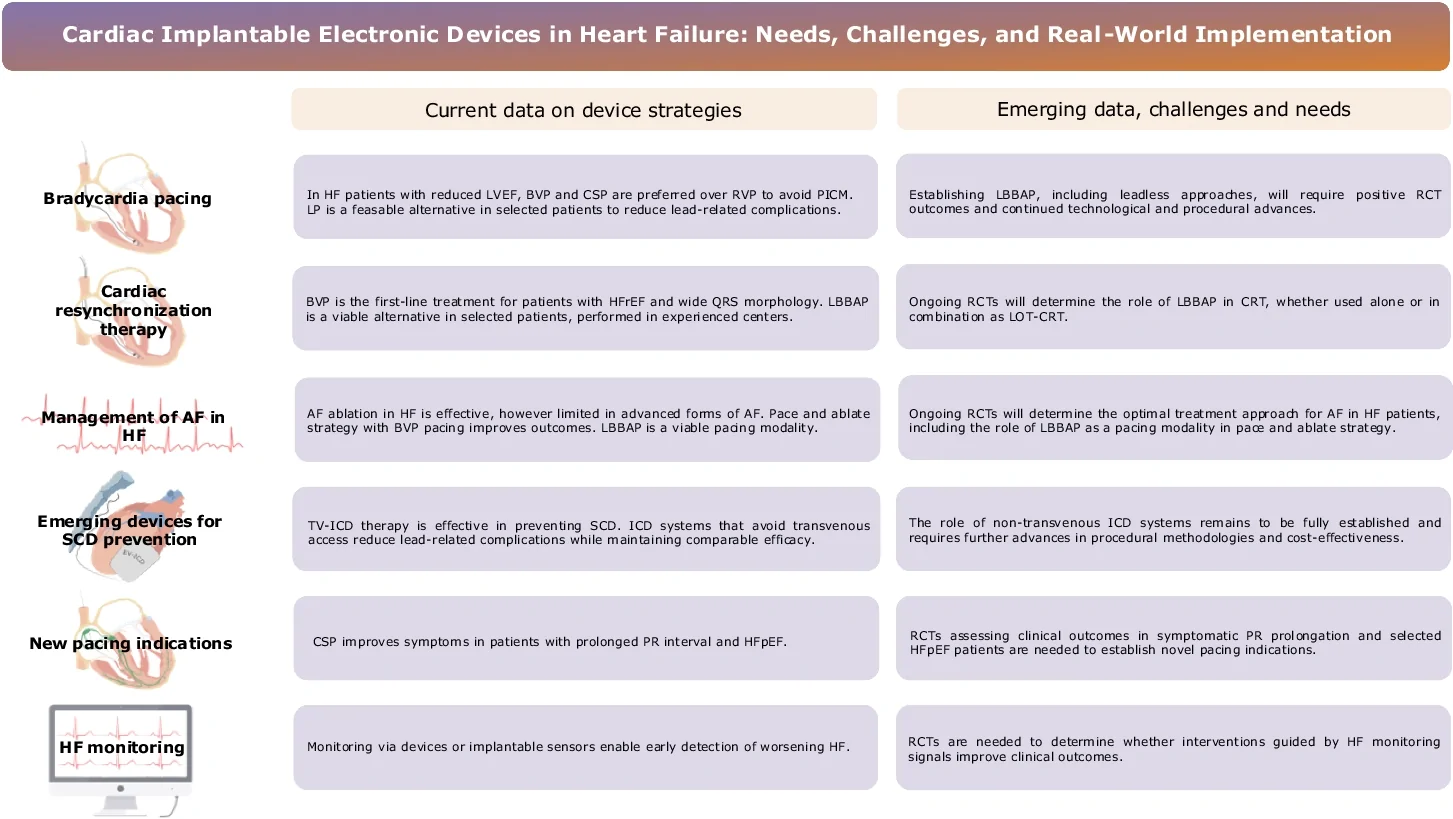

## Introduction

Cardiac implantable electronic devices (CIEDs) have undergone substantial evolution, extending well beyond traditional pacemaker therapy for arrhythmia management. In patients with heart failure (HF), CIEDs now represent a cornerstone of care, offering bradyarrhythmia treatment, cardiac resynchronization therapy (CRT), prevention of sudden cardiac death (SCD), and improvement of cardiac function. Contemporary devices increasingly incorporate sophisticated monitoring technologies that facilitate disease surveillance and optimize clinical decision-making [1]. This review highlights recent developments in CIEDs and HF, focusing on the latest scientific evidence, challenges in clinical implementation, and future directions.

## Methods

This narrative review was based on a structured literature search of PubMed/MEDLINE and contemporary international guideline and consensus documents published up to the time of manuscript preparation. Search terms included combinations of heart failure, cardiac implantable electronic devices, cardiac resynchronization therapy, conduction system pacing, left bundle branch area pacing, His bundle pacing, leadless pacing, implantable cardioverter-defibrillator, remote monitoring, and atrial fibrillation. Priority was given to contemporary clinical practice guidelines, expert consensus statements, randomized controlled trials, meta-analyses, and large prospective registries. Landmark observational studies and ongoing clinical trials were included when they addressed emerging technologies or clinical questions for which randomized evidence is currently limited. The objective was to provide a balanced overview of established evidence, recent advances, and future directions in device therapy for heart failure.

### Contemporary indications for CIEDs in heart failure

In patients with HF, CIED therapy may be indicated for the management of bradyarrhythmias, delivery of CRT, or prevention of SCD. Current evidence and clinical maturity of established and emerging CIED therapies in heart failure is summarized in Table 1.

**Table 1:** Current evidence and clinical maturity of established and emerging CIED therapies in heart failure

Device strategy	Main evidence base	Current clinical position
Resynchronization with biventricular pacing in HF	Multiple landmark RCTs demonstrating reductions in HF hospitalization and mortality	Standard of care
Biventricular pacing for prevention of PICM	Randomized trials and guideline recommendations	Standard of care
CSP for prevention of PICM	Observational studies, registries, smaller randomized trials, ongoing large RCTs	Reasonable alternative in selected patients
CSP as alternative resynchroniztion strategy	Small to medium-sized randomized trials with mixed results	Selected patients, experienced centres
CSP after failed biventricular resynchronization therapy	Observational studies and expert consensus	Established bail-out strategy
CSP optimized biventricular resynchronization (e.g. LOT-CRT)	Mechanistic studies and observational data	Emerging strategy
Leadless pacing	Large observational studies and registry data, no RTCs	Established alternative in selected patients
Leadless LV endocardial pacing (WiSE-CRT)	Feasibility studies and limited prospective clinical data	Alternative expert-centre strategy
Biventricular pacing after AVNA ablation	Randomized trials and meta-analyses	Established strategy
CSP after AVNA ablation	Observational studies, small RCTs and meta-analyses	Emerging alternative
AV-optimized pacing for symptomatic prolonged PR interval	Mechanistic studies and small randomized crossover trials	Investigational
Personalized accelerated pacing in HFpEF	Small prospective and randomized studies	Investigational
Transvenous ICD	Multiple landmark randomized trials	Standard of care
Subcutaneous ICD (S-ICD)	Large registries and comparative studies	Established alternative
Extravascular ICD (EV-ICD)	Prospective pivotal studies and post-marketing data	Emerging alternative
Modular leadless pacing-defibrillator systems	Early clinical studies	Investigational
Multiparametric device monitoring (e.g., HeartLogic)	Prospective validation studies and observational data	Emerging clinical tool
Pulmonary artery pressure monitoring (CardioMEMS)	Randomized outcome trials	Established option in selected HF patients

*HF* heart failure, *RCT* randomized clinical trial, *CSP* conduction system pacing, *PICM* pacing-induced cardiomyopathy, *AV* atrio-ventricular, *LOT-CRT* left bundle branch-optimized cardiac resynchronization therapy, *HFpEF* heart failure with preserved ejection fraction, *ICD* implantable cardioverter defibrillator

#### Bradycardia pacing

While indications for bradycardia pacing are consistent between patients with and without HF, the presence of ventricular dysfunction requires careful selection of pacing modality to avoid deterioration of left ventricular ejection fraction (LVEF) and the development of pacing-induced cardiomyopathy (PICM) [2, 3]. Accordingly, biventricular pacing (BVP) and conduction system pacing (CSP) are increasingly preferred over conventional right ventricular pacing (RVP) [4, 5]. The ESC clinical consensus statement on conduction system pacing and the HRS/APHRS/LAHRS consensus on cardiac physiologic pacing are largely concordant in emphasizing that the optimal pacing strategy depends on LVEF and the anticipated burden of ventricular pacing [4, 5]. In patients with a low expected ventricular pacing burden (< 20%) and preserved or mildly reduced LVEF (> 40%), conventional RVP with minimization algorithms is generally recommended, although CSP may be considered as an alternative physiologic pacing strategy, particularly in the setting of potential progression of conduction disease. In patients with a higher anticipated ventricular pacing burden and mildly reduced LVEF, both BVP and CSP may be considered as alternatives to RVP to reduce the risk of PICM [4, 5]. In patients with LVEF < 40%, ESC guidelines favour BVP, while CSP may be considered as an alternative, particularly when CRT delivery is challenging or when a less complex device strategy is preferred [2, 4]. Similarly, HRS/APHRS/LAHRS recommendations align these patients with CRT indications, with BVP remaining the preferred pacing modality in those with LVEF ≤ 35% [5].

#### Cardiac resynchronization

CRT is indicated in patients with symptomatic heart failure, LVEF ≤ 35%, persistent symptoms despite optimal guideline-directed medical therapy (GDMT), and a widened QRS complex Importantly, CRT should be considered as part of a comprehensive HF treatment strategy. Optimization of contemporary GDMT remains essential before device implantation whenever feasible, as pharmacological therapy may improve ventricular function, symptoms, and overall prognosis [2, 5–7]. The magnitude of benefit and strength of recommendation are primarily determined by QRS morphology and duration [5, 6, 8]. CRT is recommended in patients in sinus rhythm with left bundle branch block (LBBB) and QRS duration ≥ 150 ms. It should also be considered in those with LBBB and QRS duration 130–149 ms, as well as in patients with non-LBBB morphology and QRS ≥ 150 ms. In patients with non-LBBB morphology and QRS duration 130–149 ms, CRT may be considered, albeit with a lower expected benefit [5, 6, 9]. In patients with atrial fibrillation, CRT is recommended, with the strongest evidence in those with NYHA class III–IV symptoms and QRS ≥ 150 ms [2, 6]. Consistent with these recommendations, real-world data indicate that atrial fibrillation is not associated with reduced CRT utilization and does not modify the association between CRT and lower risks of adverse clinical outcomes [10]. Achieving a high percentage of effective biventricular pacing is important, and atrioventricular node ablation (AVNA) may be considered when adequate pacing cannot otherwise be ensured, however, recent study did not show improvement in mortality or non-fatal HF events compared to medical rate control [2, 11]. In patients eligible for CRT, CSP may be considered as an alternative to BVP, either as a primary strategy in selected patients or when coronary sinus lead placement is unsuccessful or not feasible [2, 4, 5]. Although CSP has emerged as an important physiological pacing strategy, the strength of supporting evidence varies across different clinical indications. Conventional BVP remains the standard CRT approach for most eligible patients, supported by multiple landmark randomized trials demonstrating improvements in ventricular remodeling, heart failure outcomes, and survival, as well as by contemporary guideline recommendations [2, 5, 12, 13]. Consideration is warranted in patients who develop symptomatic PICM following conventional RVP. In such cases, device upgrade to CRT using either BVP or CSP is recommended in patients on optimal GDMT with LVEF ≤ 35%, and may also be considered in those with LVEF 35–40% [2, 14].

#### Prevention of sudden cardiac death

Implantable defibrillator implantation for primary prevention is recommended in patients with symptomatic heart failure despite GDMT, ischemic cardiomyopathy, and LVEF ≤ 35%, and should be considered in comparable patients with non-ischemic cardiomyopathy. Contemporary GDMT has substantially improved outcomes in patients with HFrEF and may reduce the risk of ventricular arrhythmias and sudden cardiac death. Reassessment of left ventricular function following optimization of medical therapy therefore remains an important component of primary prevention ICD decision-making [15]. Candidates for ICD implantation should have a reasonable expectation of survival with good functional status for more than one year. In contemporary practice, ICD candidacy should additionally take into account competing non-arrhythmic mortality, frailty, major comorbidities, patient preferences, and anticipated quality-of-life benefit, particularly in older patients, those with improved LVEF, advanced heart failure, or significant non-cardiac disease [6, 9, 15, 16]. In patients with specific cardiomyopathies (e.g., hypertrophic cardiomyopathy, arrhythmogenic cardiomyopathy, or cardiac sarcoidosis) or pathogenic variants associated with a high risk of sudden cardiac death, primary prevention ICD implantation may be indicated even when LVEF is > 35%; in such cases, disease-specific recommendations, risk algorithms, and validated risk scores should be applied when available [16, 17]. Although overall indications are largely concordant, ESC guidelines assign a weaker recommendation (Class IIa) for non-ischemic cardiomyopathy compared with the Class I recommendation in American guidelines [6, 9]. For primary prevention, ICD implantation is not recommended within 40 days following myocardial infarction. It is also generally not indicated in patients with advanced heart failure and severe symptoms unless CRT or advanced HF therapies are concurrently considered [6, 16]. ICD implantation for secondary prevention is indicated in patients who have survived sudden cardiac arrest or sustained hemodynamically significant ventricular arrhythmias in the absence of a reversible cause. These indications are consistent across contemporary European and American guidelines [17, 18].

### Current evidence for pacing to prevent heart failure and conventional pacemaker-related complications

**Fig. 1:**
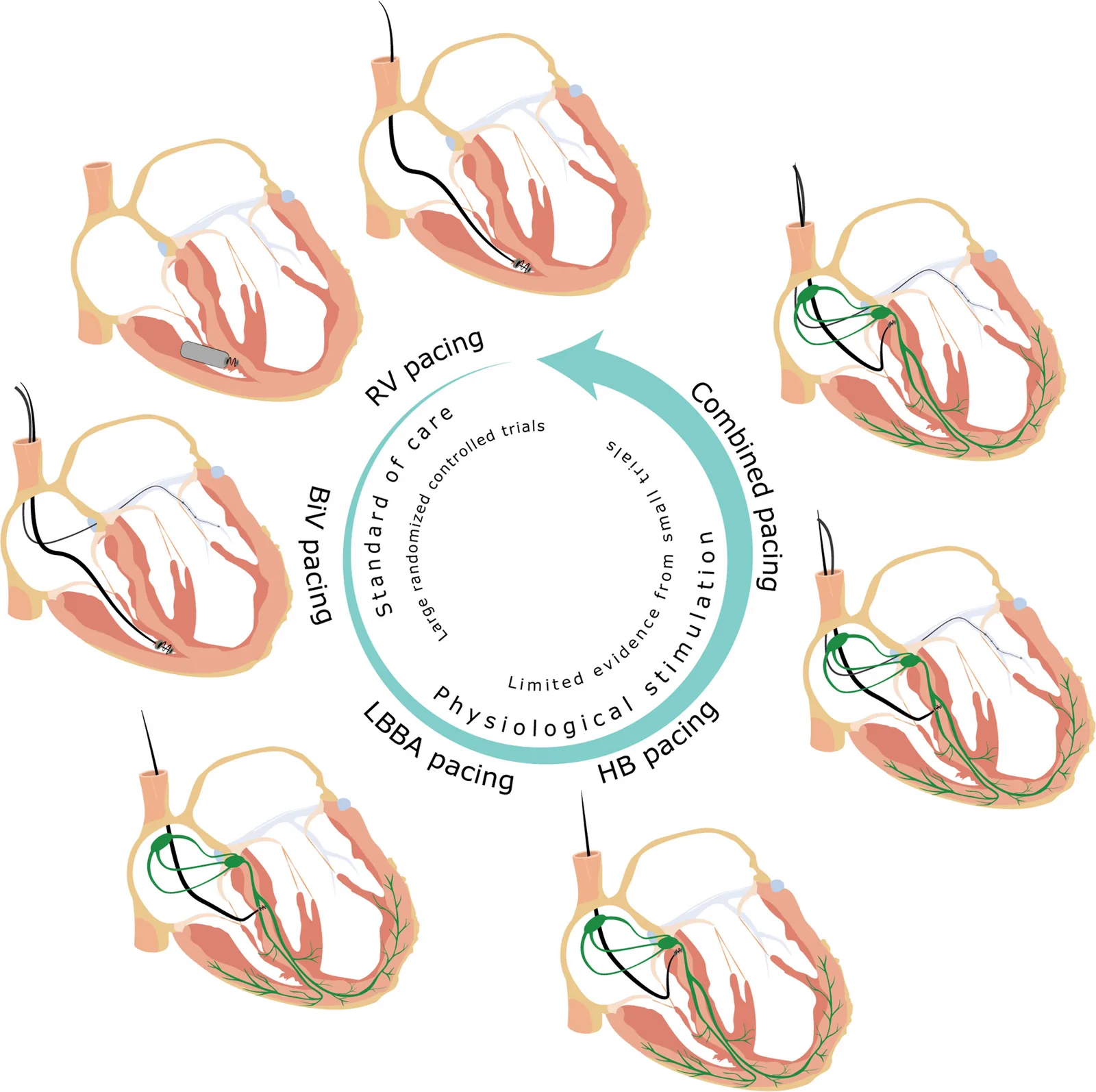
Pacing modalities in heart failure. Evolution of pacing and resynchronization modalities for prevention and treatment of heart failure. RV pacing—right ventricular pacing using leadless device or transvenous lead, BiVP pacing—biventricular pacing, HB pacing—His bundle pacing, LBBAP pacing—left bundle branch area pacing, Combined pacing – left bundle branch optimized cardiac resynchronization therapy (LOT-CRT) and his bundle optimized cardiac resynchronization therapy (HOT-CRT)

Although conventional RVP effectively prevents bradyarrhythmias, implantation of transvenous (TV) pacemakers is associated with important long-term risks. Approximately 10% of patients experience complications within 5 years after conventional pacemaker implantation [19]. These complications can be broadly categorized as lead-related, device-related, and PICM. To reduce lead- and device-related complications, leadless pacemakers (LPs) were developed. Although randomized comparisons between conventional TV pacemakers and LPs remain limited, observational studies suggest that LPs may reduce device-related reinterventions, infections, and possibly even HF hospitalizations [1, 20]. However, LP implantation has also been associated with a higher risk of cardiac perforation compared with conventional pacemaker implantation, as well as suboptimal atrioventricular synchrony [21, 22]. An important long-term complication of non-physiological pacing is PICM. The reported incidence varies considerably, ranging from 6 to 25%, largely because of the absence of a standardized definition, uncertainty regarding the threshold defining a high RV pacing burden (typically ranging from 20 to 40%), and the lack of distinction between pacing-induced and pacing-related cardiomyopathy [3, 5]. Most studies define PICM as a decline in LVEF to < 50%. Strategies to mitigate PICM include BVP, which requires an additional coronary sinus lead, and CSP, in which the ventricular lead is positioned at the His bundle or within the left bundle branch area [23–25]. Current recommendations favour BVP over CSP in HF patients, primarily based on the results of the BLOCK HF randomized trial, which demonstrated that among patients with HF, LVEF ≤ 50%, and an anticipated high RV pacing burden, BVP was superior to RVP across several clinical outcomes [4, 26]. However, these findings were not confirmed in the BioPace trial, which predominantly enrolled patients with preserved LVEF and narrow QRS complexes [27]. More recently, CSP technique, particularly left bundle branch area pacing (LBBAP), which is technically more feasible than His bundle pacing (HBP), has emerged as a promising physiological pacing strategy capable of preserving or restoring near-normal ventricular activation [5] (Fig. 1). Evidence supporting LBBAP over RVP is currently derived mainly from observational studies suggesting improved survival in patients with a pacing indication and a high anticipated pacing burden [28–30]. Several smaller randomized trials have demonstrated better preservation of cardiac function with LBBAP, although they have not shown significant reductions in HF hospitalizations or mortality [31–33]. As implantation techniques and dedicated tools continue to evolve, the results of ongoing large, randomized trials (Table 1 and 2) and further refinements in procedural methodology will be crucial for the safe and effective implementation of LBBAP across centers with varying levels of expertise [37].

**Table 2:** Ongoing randomized trials evaluating conduction system pacing

	**NCT number**	**Clinical scenario**	**LVEF criterion**	**Planned N**	**CSP modality**	**Comparator**	**Primary endpoint**	**Estimated completion/reporting**	**Status/comment**
RVP/minimized VP comparator									
PROTECT-HF	NCT05815745	Bradycardia requiring ventricular pacing	LVEF > 35%	2600	CSP (HBP or LBBAP)	RVP	Death and unplanned HF acute care (HF hospitalization or ambulatory IV diuretic therapy)	Primary completion ~ 2029; reporting 2030–2031	Large hard-endpoint trial; recruiting
PhysioVP-AF [34]	NCT05367037	SND or paroxysmal AV block with prolonged PR interval	Excludes CRT/ICD candidates	640	CSP (HBP or LBBAP)	RVP with managed ventricular pacing	Occurrence of persistent AF	Completion ~ 2028; reporting 2029–2030	Active recruiting; comparator is minimized ventricular pacing rather than high-burden RVP
PHYSPAVB	NCT05214365	AV block requiring pacing	LVEF > 50%	200	CSP (HBP or LBBP)	RVP	PICM or admission due to HF at 12 months	2026–2027	Ongoing/unpublished
BOSTON PACE	NCT05869500	High-grade AV block with anticipated > 40% VP	LVEF > 50%	100	LBBAP	RVP	Change in LVEF and LVESV at 12 months	2026–2027	Pilot feasibility trial. Recrution completed
PROTECT-SYNC	NCT05585411	Bradyarrhythmia with anticipated > 40% VP	Excludes CRT/ICD candidates	450	LBBAP	RVP	Composite of all-cause death, HFH, PICM and CRT upgrade at 2 years	2027–2028	Recruiting, estimated completion 2026
LEAP-Block	NCT04730921	AV block with anticipated > 40% VP	LVEF > = 50%	486	LBBAP	RVP	Time to first event: all-cause death, HFH, or device upgrade due to HF within 2 years of implantation	2027–2028	Completed
LEAP	NCT04595487	AV conduction disorder; expected VP > = 20%	LVEF > = 40%	470	LVSP	RVP	Composite of death, HF hospitalization, or > 10% absolute LVEF decrease leading to LVEF < 50% at 1 year	Completion likely 2025; reporting 2026?	Unknown status
OptimPacing	NCT04624763	AV block/brady pacing	LVEF > 35%	683	LBBP	RVP	Composite of all-cause death, HFH, and/or PICM at 6, 12, 24 and 36 months	Likely 2028	Active, not recruiting
LEFT-HF	NCT05015660	High-degree AV block; anticipated VP > 90%	LVEF > 35%	1300	LBBP	RVP	Time to CV death, first HF event at 36 months and time to worsening LVESVi at 24 months	Completion ~ 2026; reporting 2030	Recruiting
PROTECT-UP	NCT06052475	Upgrade from chronic RVP in mild-moderate LV impairment	LVEF 35–50%	155	CSP (HBP or LBBP); BVP if CSP not deliverable	Continued RVP	SF-36 physical component score under blinded crossover conditions	2027–2028	Active/recruiting; crossover design
NOVEL II	NCT07464041	AV block with anticipated > = 40% VP	LVEF > 50%	200	LBBAP with stylet-driven lead	RVP	Change in LVEF at 12 months	2027–2028	Recruiting
LBBAP vs MVP	NCT07314008	Sick sinus syndrome with prolonged PR interval (> 200 ms)	LVEF > 35%	440	LBBAP	RVP with managed ventricular pacing	Composite of all-cause mortality, HFH, and persistent AF at 2 years	2029	Recruiting
BVP/CRT comparator									
SYNCHRONICITY/Boston Scientific LBBAP-CRT	NCT07069738	De novo CRT-D indication in HF with LBBB	LVEF < = 35%	850	LBBAP	BVP	Composite effectiveness endpoint including death, transplant, LVAD, HF events, VT/VF requiring device intervention up to 5 years; safety endpoint, system-related complication-free rate at 12 months	2029–2030	Industry-sponsored trial; enrolling
LEFT vs LEFT	NCT05650658	HF with LVEF < = 50% and wide QRS or current/anticipated high VP burden	LVEF < = 50%	2136	CSP (HBP or LBBAP)	BVP	Composite of all-cause mortality and HF hospitalization (5,5 years FU)	Primary completion ~ 2029; reporting 2030	Recruiting
CONSYST-CRT II	NCT06105580	Symptomatic HF with CRT indication or brady pacing indication	LVEF < = 35% for CRT LVEF < 40% for brady	320	CSP (HBP or LBBAP)	BVP	Composite of all-cause mortality, cardiac transplant, or HF hospitalization at 12 months	Primary completion Nov 2027; reporting 2028	Larger clinical-endpoint successor to CONSYST-CRT
HIS-ALT II	NCT04409119/NCT05814263	Symptomatic HF with typical LBBB (CRT indication) or need for upgrade of existing device	LVEF < = 35%	150	CSP (HBP or LBBAP)	BVP	Change in LVESV/feasibility of QRS narrowing	Reporting likely 2027	Main trial plus UK-site registration. Completed 2026
HIS-CRT	NCT05265520	HF with RBBB requiring CRT	LVEF < = 35%	120	HBP	BVP	Change in LVEF at 6 months	2027–2028	RBBB-specific CRT trial. Recruiting
LeCaRT	NCT05365568	CRT-indicated HF	LVEF < 40%	170 planned; 168 randomized	LBBAP	BVP	Composite: death, HF hospitalization, device-related reintervention, or failure to deliver assigned CRT	Presented 2026; full paper likely 2026–2027	Completed
NORDIC-CSP	NCT07492979	CRT, upgrade or brady pacing indication	LVEF < = 40%	1100	CSP (HBP or LBBAP)	BVP	Composite of time to death or first non-planned HF hospitalisation	2031	New 2026 record; not yet recruiting
DA VINCI	NCT07107048	CRT indication, LBBB	LVEF < = 35%	194	LBBAP	BVP	CRT response: freedom from cardiac death, HFH, ≥ 15% relative reduction in left ventricular end-systolic volume	Completion estimated 2027	Not yet recruiting
CSP vs surgical LV	NCT06342492	Patients that underwent CRT with BVP and failed CS implantation	Not specified (LVEF < 40%?)	100	CSP	transthoracic epicardial LV lead placement	Need for lead revision	Estimated completion 2024?	Recruiting
BATTLE	NCT06061627	Patients with HF, CRT indication and IVCD	LVEF < = 35%	86	LOT-CRT	BVP	Change in LVEF at 6 months	Estimated completion 2027	Recruiting
ACHIEVED LBBAP	NCT07526896	Patients with HF, CRT indication, and LBBB (Strauss criteria)	LVEF < = 35%	80	LBBAP	LOT-CRT	Change in LVEF and LVESV at 6 and 12 months; crossover design	Estimated completion 2029	New, not yet recruiting, Industry sponsored
AF/pace-and-ablate or non-pacing comparator									
CONDUCT-AF	NCT05467163	HF + symptomatic AF, narrow QRS, pace & ablate strategy	LVEF < 50%	82	CSP (HBP or LBBAP)	BVP	Change in LVEF at 6 months	Presented 2026; full paper likely 2026	Completed
APAF-CSP	NCT07604727	Symptomatic HF + AF unsuitable for rhythm control; pace & ablate strategy	Across EF	292	CSP (HBP or LBBAP)	BVP	Hierarchical composite: all-cause mortality, HF hospitalization/urgent HF visit, and > = 5-point KCCQ improvement (mean FU 2,5 years)	New 2026 registration; likely 2030 +	Broad pace-and-ablate implementation trial
PACE-FIB [35]	NCT05029570	Permanent AF + HF	LVEF > 40%	334	CSP + AVNA	Optimal medical/pharmacological therapy	Composite of all-cause mortality, HFH, and worsening HF (36 months)	2030	Recruiting
RAFT-P&A	NCT06299514	Symptomatic HF with permanent/persistent AF; pace & ablate strategy	Across EF	600	CSP + AVNA	Optimal medical/pharmacological therapy	Composite/hierarchical objective including mortality, HF hospitalization, NT-proBNP and QoL (Winratio)	Completion 2029, reporting 2030–2031	Recruiting
ABACUS [36]	NCT06207383	Persistent AF with HF; eligible for AF ablation or CSP + AVNA	Across EF	220	CSP + AVNA	AF catheter ablation/PVI	Co-primary endpoints: superiority for mortality + CV hospitalization and non-inferiority for mortality + HF hospitalization (1–4 years)	Completion 2028; reporting 2029	Recruiting
AVENUE PACE	NCT07059208	Persistent AF > 65 years or paroxysmal AF > 75 years, HR > 70bpm on resting ECG, failed pharmacological therapy	Across EF	172	CSP + AVNA	PVI	All cause rehospitalisation (2 years)	Completion 2029; reporting 2031	Not yet recruiting
AVA CONDUCT	NCT07428967	Symptomatic AF with scheduled AVNA	LVEF = > 50%	82	LBBAP + AVNA	RVP + AVNA	No. developing PICM at 36 months	Completion 2028; reporting 2029	Recruiting
HF-RELIEF	NCT06833138	Permanent AF, = > 1 HF hospitalisation in previous year, narrow QRS, average HR < = 110 bpm	= > 50%	266	CSP + AVNA	Optimal medical/pharmacological therapy	Time to composite of all-cause mortality, HF hospitalisation or IV diuretics (24 months)	December 2029	Recruiting
HFpEF									
ELEVATE-HFpEF	NCT06678841	Symptomatic HFpEF without an established pacing indication; personalized accelerated pacing	= > 50%	700	Personalized accelerated pacing	Pacing OFF	Time to the composite of all-cause death, HF hospitalization, or HF worsening requiring IV diuretics at 12 months	Completion 2029	Recruiting

The table summarizes ongoing, recently completed, or not-yet-fully-published randomized trials evaluating conduction system pacing in bradycardia pacing, cardiac resynchronization therapy, upgrade procedures, and pace-and-ablate strategies. Trials are grouped by comparator: right ventricular pacing/minimized ventricular pacing, biventricular pacing/cardiac resynchronization therapy, and atrial fibrillation/pace-and-ablate or non-pacing comparators. Trial characteristics, LVEF criteria, planned sample size, pacing modality, comparator, primary endpoint, estimated reporting timeframe, and status are shown. Trial details should be verified against the original registry record before manuscript submission

*AF* atrial fibrillation, *AV* atrioventricular, *AVNA* atrioventricular node ablation, *BVP* biventricular pacing, *CRT* cardiac resynchronization therapy, *CSP* conduction system pacing, *CV* cardiovascular, *EF* ejection fraction, *FU* follow-up, *HBP* His bundle pacing, *HF* heart failure, *HFH* heart failure hospitalization, *ICD* implantable cardioverter-defibrillator, *KCCQ* Kansas City Cardiomyopathy Questionnaire, *LBBAP* left bundle branch area pacing, *LBBB* left bundle branch block, *LBBAP* left bundle branch pacing, *LV* left ventricular, *LVAD* left ventricular assist device, *LVEF* left ventricular ejection fraction, *LVESV* left ventricular end-systolic volume, *LVESVi* left ventricular end-systolic volume index, *LVSP* left ventricular septal pacing, *MVP* minimized ventricular pacing, *NCT* National Clinical Trial number, *NT-proBNP* N-terminal pro-B-type natriuretic peptide, *PICM* pacing-induced cardiomyopathy, *PVI* pulmonary vein isolation, *QoL* quality of life, *RBBB* right bundle branch block, *RVP* right ventricular pacing, *SND* sinus node dysfunction, *VF* ventricular fibrillation, *VP* ventricular pacing, *VT* ventricular tachycardia

### Current evidence for devices in cardiac resynchronization therapy

Cardiac resynchronization therapy is an established treatment for patients with HF, reduced LVEF, and LBBB. Traditionally, CRT has been synonymous with BVP. Several studies and large randomized clinical trials have consistently demonstrated that CRT improves cardiac function, promotes reverse ventricular remodelling and improves outcomes [12, 13, 38, 39]. Despite robust evidence supporting its efficacy, BVP has some limitations. These include anatomical constraints of the coronary venous system that may hinder optimal lead placement, phrenic nerve stimulation, and potentially incomplete electrical resynchronization with increased risk of arrhythmias [40–43].

To overcome some of the limitations associated with conventional BVP, leadless LV endocardial pacing has been developed as an alternative resynchronization strategy. The WiSE-CRT system (Wireless Stimulation Endocardially for Cardiac Resynchronization Therapy) delivers ultrasound-mediated energy from a subcutaneously implanted transmitter and battery to a leadless LV endocardial electrode, enabling physiological LV endocardial stimulation without the need for a coronary sinus lead. Despite the proven feasibility of this approach, widespread clinical adoption has been limited by several important drawbacks, including high device costs, notable complication rates, and the potential need for prolonged anticoagulation [44]. Several smaller randomized studies conducted in experienced centers demonstrated non-inferiority of LBBAP compared with BVP in CRT candidates. However, these trials primarily assessed surrogate endpoints, such as echocardiographic reverse remodelling and QRS narrowing, rather than hard clinical outcomes [45–47]. In contrast, more recent multicenter randomized trials assessing hard clinical outcomes alongside surrogate measures have yielded conflicting results. The HeartSync-LBBP randomized trial, which enrolled 200 CRT patients across six experienced centers in China, demonstrated superiority of LBBAP over BVP for the composite endpoint of all-cause mortality and HF hospitalization when a highly selective criteria of LBBB correctable by His bundle pacing was used in the LBBAP arm, thus identifying a specific subgroup of patients with LV “dyssynchronopathy” [48]. In contrast, two similarly sized multicenter trials failed to demonstrate non-inferiority of LBBAP compared with BVP [49, 50]. In the Brazilian PhysioSync-HF trial, conducted across 14 centers where only 57% of operators had experience with more than 40 CSP procedures, CSP was inferior to BVP with respect to the hierarchical primary endpoint of all-cause mortality, HF hospitalization, and LVEF improvement [49]. Although several factors may have contributed to these findings, including a heterogeneous patient population and crossover between treatment groups, differences in CSP technique likely played a major role, as only 69% of patients received LBBAP in PhysioSync-HF compared with 98% in HeartSync-LBBP. Similarly, the Spanish multicenter LEFT-BUNDLE CRT trial failed to demonstrate non-inferiority of LBBAP versus BVP; however, the exceptionally high CRT response rate (defined either by an improved clinical composite score or ≥ 15% reduction in left ventricular end-systolic volume at 6-month follow-up) in the BVP arm and a relatively high crossover rate (20.7%) may have limited the ability to establish non-inferiority of this novel pacing modality [50]. Moreover, about 50% of patients were classified as having LV septal pacing rather than conduction system engagement, thus making the clinical value of true LBBAP difficult to assess. However, it should be noted that contemporary randomized CRT trials have set the benchmark for CRT echocardiographic response at 77–80% and clinical response at 90% (all-cause mortality < 4% yearly, CV hospitalizations < 10% yearly) [12, 13, 38, 39]. Thus, while the LEFT-Bundle CRT trial outcome was reasonably expected, the 28% HF hospitalizations rate observed in the HeartSync-LBBP [48] CRT arm remains unexplained (4 –fold higher compared to the literature) and possibly reflects selection bias in light of the 90% correctability of LBBB observed in the LBBAP arm. As guidelines and consensus documents increasingly endorse CSP as an alternative to conventional BVP [4, 5], these mixed findings highlight the need for cautious and selective implementation. Differences among contemporary randomized trials likely reflect a combination of methodological and clinical factors, including patient selection, baseline degree of conduction system disturbances resulting in LBBB, operator experience, definitions of conduction system capture versus left ventricular septal pacing, crossover rates, endpoint selection, and follow-up duration. Importantly, these discrepancies may also reflect genuine uncertainty regarding whether improved electrical resynchronization consistently translates into superior clinical outcomes across all CRT-eligible patients. Ongoing and future multicenter randomized trials (Table 2) with standardized implantation criteria, rigorous confirmation of conduction system capture, and long-term clinical endpoints will be essential to better define the role of CSP in CRT candidates. Ultimately, reconciling these divergent findings may refine patient selection and optimize CRT delivery, potentially through combined approaches such as left bundle branch optimized CRT (LOT-CRT) [51] (Fig. 1).

### Treatment strategies and management of atrial fibrillation in heart failure

Atrial fibrillation (AF) is characterized by disorganized atrial activity and an irregular, rapid ventricular response, leading to reduced cardiac output (CO) and hemodynamic impairment [52–55]. Restoration of ventricular rate control and regularity can improve hemodynamics and symptoms. Two main therapeutic strategies are available: rhythm and rate control. Rhythm control can be achieved with antiarrhythmic drugs and/or catheter ablation (CA), aiming to restore sinus rhythm (SR) and atrial contribution to CO. In patients with HF, CA has shown superiority over GDMT therapy in improving outcomes, with the CASTLE-AF trial demonstrating reductions in mortality and hospitalizations for worsening HF [56–59]. Accordingly, current European and US guidelines recommend CA as first-line therapy in HF patients with AF [60, 61]. However, the success rates of rhythm control strategies in patients with persistent forms of AF are limited [62, 63]. Data in patients with recurrent AF, the elderly, and those with multiple comorbidities remains scarce, particularly regarding the risks of repeat procedures and associated complications. In this subset of patients who cannot be adequately rate-controlled with medical therapy and in whom rhythm control has failed or is not appropriate, atrioventricular node ablation (AVNA) followed by pacemaker implantation (‘pace-and-ablate’) offers a safe and effective alternative, ensuring reliable rate control, regularization of ventricular rhythm, elimination of rate-control medications, and rapid symptomatic relief [64]. The choice between catheter ablation and a pace-and-ablate strategy should be individualized according to the underlying AF phenotype and likelihood of successful rhythm control. Catheter ablation should be prioritized in patients with AF-mediated cardiomyopathy or a high likelihood of maintaining sinus rhythm, whereas ‘pace-and-ablate’ strategy represents a reasonable alternative in older patients with long-standing persistent or permanent AF, multiple comorbidities, failed rhythm-control attempts, or inadequate rate control. A recent meta-analysis evaluating five principal treatment strategies for AF found that CA was most effective in preventing AF recurrence, whereas the pace-and-ablate approach was associated with lower mortality and fewer rehospitalizations [65]. Current ESC and US guidelines recommend AVNA in patients with refractory rapid ventricular response who are unresponsive or intolerant to intensive rate and rhythm control therapy, and not eligible for rhythm control by CA (Class IIa, Level of evidence B) [60, 61]. The management pathway therefore involves two sequential decisions: whether catheter ablation is likely to achieve durable rhythm control and, if a pace-and-ablate strategy is pursued, selecting the pacing modality that best preserves physiological ventricular activation and long-term ventricular function, as pacemaker dependency makes this choice a key determinant of clinical outcomes.

When combined with AVNA, RVP has been shown to improve several clinically relevant outcomes, including symptoms, quality of life, exercise capacity, and LVEF [66]. However, the principal long-term concern after AVNA is pacing-induced dyssynchrony from conventional RVP, which can precipitate or worsen preexisting HF [64, 67, 68]. The deleterious effects of RVP can be mitigated by BVP, which, according to randomized trials, including APAF-CRT study, and meta-analyses, when combined with AVNA, leads to reductions in mortality, HF hospitalizations, and adverse LV remodelling [69–71]. Therefore, ESC guidelines provide a Class I recommendation for BVP in patients with reduced LVEF undergoing AVNA, and a Class IIa recommendation in those with mildly reduced LVEF, whereas US guidelines assign a Class IIa recommendation in patients with reduced LVEF < 50% to avoid pacing-induced dyssynchrony [2, 5]. However, BVP does not fully restore physiological activation and remains associated with QRS prolongation and residual electrical and mechanical dyssynchrony [72].

CSP is a feasible alternative to conventional RVP and BVP. Compared to initial experience with HBP, LBBAP is less affected by the ablation site, shows more stable long-term parameters without the need of a RV back-up lead, and therefore represents a more practical physiological alternative [73]. Several observational studies have already reported greater symptomatic and echocardiographic improvement with CSP after AVNA, compared with BVP, particularly in those with narrow baseline QRS and reduced EF [74, 75] To date, the only randomized study in this setting, the ALTERNATIVE-AF trial, proved significant improvement in LVEF with HBP compared with BVP in patients with persistent AF, reduced LV function, and narrow QRS [76]. A recent meta-analysis further suggests that CSP achieves narrower QRS duration with similar clinical and echocardiographic outcomes and no survival benefit over BVP [77]. Data in patients with preserved EF and no HF diagnosis are lacking. European And US pacing guidelines consider a Class IIb alternative to BVP in patients undergoing AVNA, with no formal recommendation for LBBAP at that time, whereas the latest 2025 ESC consensus statement provides a stronger endorsement of CSP use across all EF ranges to preserve or improve LV function and HF outcomes [2, 4, 5]. Several ongoing trials (Table 2) will further illuminate the role of pace-and-ablate strategy in HF patients with AF, including randomized comparisons of pace-and-ablate with CSP versus medical rate control (RAFT P&A trial), head-to-head comparisons of CSP versus BVP following AVNA (CONDUCT-AF trial), and comparisons of CA versus pace-and-ablate strategy (ABACUS trial). However, definitive randomized evidence from these studies is still awaited [36, 78].

### Current and emerging devices for prevention of sudden cardiac death in heart failure

SCD rate in HF patients due to systolic dysfunction is substantially unchanged despite GDMT [79]. SCD prevention strategy stands on trials adopting TV-ICD devices and no comparison of TV vs non-transvenous ICDs is available [80–83]. Thus, inference must be taken by the subgroups of patients with HF (EF < 35%) enrolled in non-TV-ICD studies. Subcutaneous ICD (S-ICD) offers superior immunity by lead-related complications compared to TV-ICD, albeit suffering the lack of ATP, which can be significantly helpful for painless monomorphic VT termination [84, 85]. S-ICD has substantial data available proving a similar efficacy to TV devices for the termination of life-threatening ventricular arrhythmias (VA) in HF patients: 3 large registries report similar efficacy, complication rate, and inappropriate shock rate compared to TV devices [86–88]. However, only one study reported a real-life comparison of S-ICD and TV-ICD recipients in a mixed population with high prevalence of HF and a mean age above 70 years, showing similar all-cause mortality, all-cause and cardiovascular readmissions, and reoperation rate with or without device infection. Thus, it appears that the S-ICD is neither burdened by device-deficiency nor offers any significant advantage in a real-life HF population. In this respect, a modular system combining a leadless unit to deliver ATP with the S-ICD has been developed, reporting a 61% efficacy to terminate clinical arrhythmias by ATP. This reinforces the approach to provide a comprehensive technology to treat VA, rather than shock-only devices [89]. However, the timing and opportunity of this modular approach is highly debatable, as recurrent MVT in S-ICD recipients may be more likely treated by VT ablation and/or antiarrhythmic drugs rather than by addition of a leadless unit to deliver ATP [90].

The extravascular ICD (EV-ICD) addresses this task owing to lead placement in the anterior mediastinum to reliably detect VAs, enabling termination either by ATP or shock [91]. In the post marketing registry, the EV-ICD has demonstrated efficacy and inappropriate shock rate similar to the S-ICD [92]. Though rapidly spreading in the clinical use, HF patients are underrepresented in the 2 largest publications, accounting for about 30% of patients [92, 93]. The advantage of the EV-ICD stands in its smaller size and extended longevity compared to the S-ICD, which makes it fit also for paediatric and small-habit patients [94]. Notably, EV-ICD is a reliable alternative for patients unsuitable to an S-ICD but is contraindicated in patients with previous sternotomy.

Both the S-ICD and the EV-ICD offer the unique possibility of a modular approach to HF patients who firstly require the SCD prevention strategy, than develop indication to either CRT or brady-arrhythmia treatment: a number of case series or case reports has demonstrated the safe coexistence of non-communicating devices implanted in the same patient with an S-ICD without untoward interferences: either TV, leadless and epicardially-implanted leads [95]. CSP co-implanted with an S-ICD has also proven to be feasible in the complex scenario of HBP. Non-communicating CRT and leadless pacemakers have been implemented safely with the EV-ICD, and in-silico model do not show significant interaction [96].

**Fig. 2:**
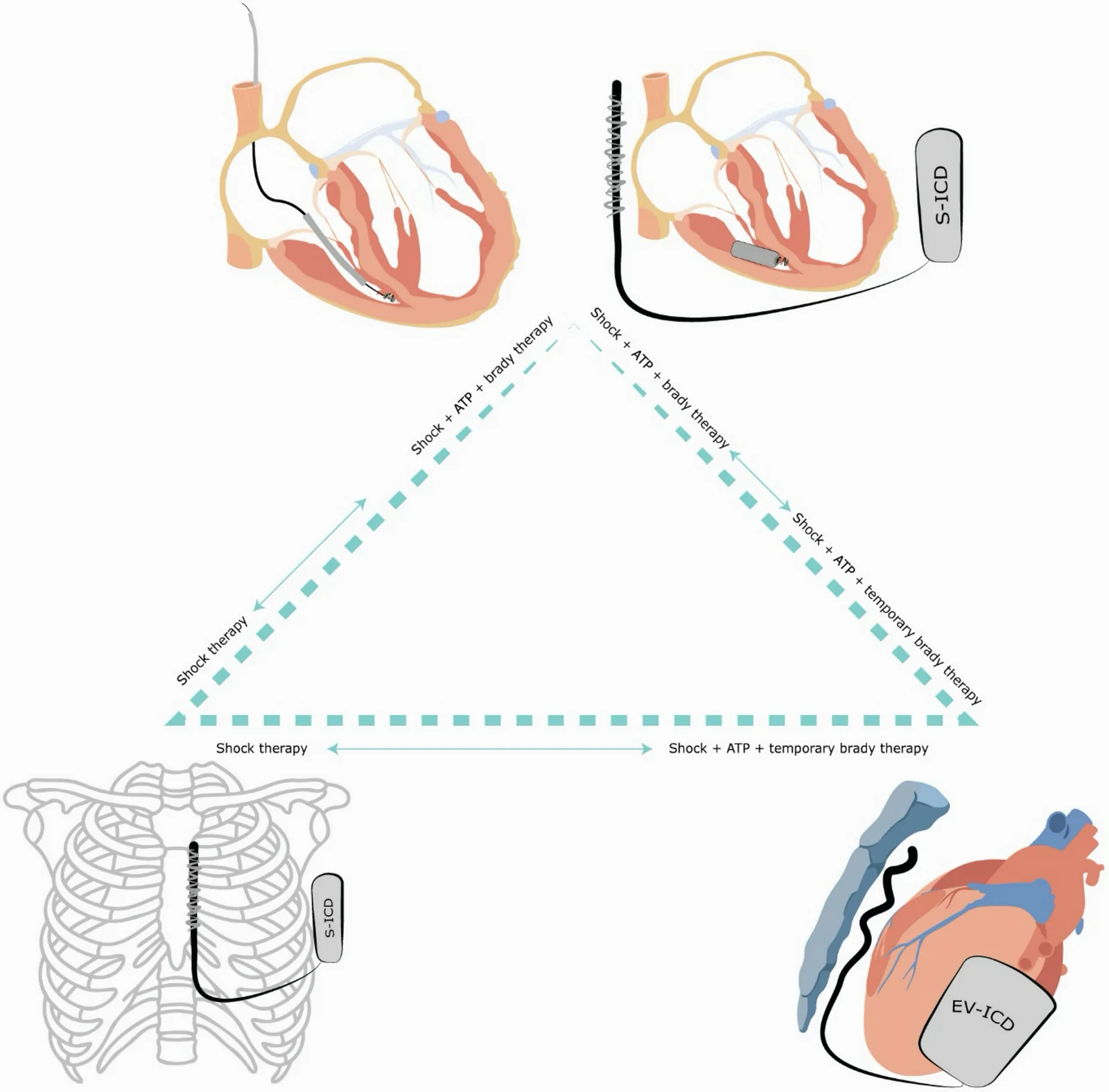
Sudden cardiac death prevention therapies in heart failure. Spectrum of current devices used for the prevention of sudden cardiac death in patients with heart failure. Transvenous implantable cardioverter-defibrillator – TV-ICD, subcutaneous ICD—S-ICD, extravascular ICD—EV-ICD and modular S-ICD with leadless pacemaker for antitachycardia pacing (ATP) delivery

Eventually, owing to the availability of leadless pacemakers and CRT (possibly of CSP), non-TV-ICD offer the ideal platform for defibrillation backup in totally leadless bradyarrhythmia treatment, CRT, and even CSP [97, 98]. This approach aims to minimize the amount of intravascular burden and/or to overcome the challenges and the risks of system upgrading [99]. In the contemporary setting, individualized treatment by communicating or non-communicating devices is feasible depending on patient’s profile at presentation (Fig. 2). Comparison of implantable cardioverter-defibrillator systems with considerations for clinical decision-making is summarized in Table 3.

**Table 3:** Comparison of implantable cardioverter-defibrillator systems with considerations for clinical decision-making

	**Transvenous ICD** **(TV-ICD)**	**Subcutaneous ICD** **(S-ICD)**	**Extravascular ICD** **(EV-ICD)**	**Modular ICD** **(mCRM)**
Lead location	Transvenous (RV ± RA)	Subcutaneous, parallel to sternum	Substernal (extravascular)	Combination of EV-ICD and leadless pacemaker
Vascular access required	Yes	No	No	Yes
Defibrillation energy	20–40 J	Up to 80 J	~ 40 J	~ 40 J
ATP capability	Yes	No	Yes	Yes
Bradycardia pacing	Yes	Limited post-shock only	Temporary post-shock pacing	Full leadless pacing
VT management	ATP and shocks	Shocks only	ATP and shocks	ATP and shocks
Lead extraction complexity	Moderate–high	Low	Expected lower than TV leads	Unknown
Risk of bloodstream infection/endocarditis	Highest	Very low	Low	Very low
Risk of pneumothorax	Yes	No	No	No
Risk of venous occlusion	Yes	No	No	No
Lead fracture risk	High	Low	Unknown	Unknown
Battery longevity	7–12 years	8–10 years	Unknown	Unknown
Ideal candidates	Need for pacing, ATP, or CRT	Young patients without pacing indication, high infection risk or difficult venous access	Need ATP but wish to avoid transvenous leads	Patients requiring both defibrillation and pacing without transvenous leads
Anatomical considerations	Usually not limiting	Challenging in very low BMI or unfavorable thoracic anatomy	Substernal anatomy and prior thoracic surgery may influence suitability	Similar considerations as EV-ICD and leadless implantation
Likelihood of future CRT requirement	Preferred	Less suitable	Less suitable	Less suitable (no integrated CRT)
Current evidence base	Standard of care with the broadest evidence across HF populations	Established for appropriately selected patients without pacing indication	Early clinical experience; long-term comparative data remains limited	Early clinical experience; evidence remains limited

*ATP* antitachycardia pacing, *CRT* cardiac resynchronization therapy, *EV-ICD* extravascular implantable cardioverter-defibrillator, *ICD* implantable cardioverter-defibrillator, *J-joules* mCRM-modular cardiac rhythm management, *RA* right atrium, *RV* right ventricle, *S-ICD* subcutaneous implantable cardioverter-defibrillator, *TV-ICD* transvenous implantable cardioverter-defibrillator, *VT* ventricular tachycardia

### New indications for treatment of heart failure with cardiac implantable electronic devices

#### Pacing in prolonged PR interval

First degree AV block has traditionally been regarded as a benign finding. However, large population-based studies have demonstrated that PR interval prolongation is associated with an increased risk of AF, pacemaker implantation, HF, and cardiovascular death [100, 101]. Mechanistically, PR prolongation may impair ventricular filling by fusing diastolic phases and promoting diastolic mitral regurgitation, thereby reducing end-diastolic volume and producing HF–like symptoms. This condition, previously termed pseudo-pacemaker syndrome, is now more accurately described as AV dromotropathy [102].

Current guidelines recommend pacing in symptomatic patients with marked PR prolongation (> 300 ms) (Class IIa, Level of Evidence C), without specifying pacing modality or LVEF [2]. Early studies using RVP in this setting yielded limited benefit [103, 104]. In contrast, sub-analyses of CRT trials showed that PR prolongation identifies a higher-risk subgroup, with increased rates of HF progression and mortality; notably, this excess risk appeared attenuated following CRT, suggesting that optimisation of AV coupling may improve outcomes [105] [106]. A combined computational and clinical study reported a 10–15% increase in cardiac output with restoration of AV coupling with unchanged ventricular activation; however, this benefit was attenuated by RVP-induced ventricular dyssynchrony, supporting the use of CSP in this setting [107]. A randomized cross-over study comparing AV-optimised HBP with back-up pacing in patients with prolonged PR and reduced LVEF failed to demonstrate an increase in peak oxygen uptake but was associated with better quality of life and patient preference for active pacing [108]. In patients with preserved LVEF, AV-optimised His bundle pacing has been associated with symptomatic improvement, increased stroke volume and a trend toward improved exercise capacity [109]. Patient selection remains challenging, as the degree of PR prolongation alone appears to be a poor predictor of response [110, 111]. Given the high prevalence of first-degree AV block—1–2% in the general population and up to 50% among CRT candidates—there is substantial therapeutic potential. Larger outcome-driven studies (Table 1 and 2) are needed to define the role of pacing for AV recoupling.

#### Pacing in heart failure with preserved ejection fraction

In contrast to HF with reduced ejection fraction, where beta-blockers and heart rate reduction improve outcomes, this strategy has not shown benefit in HF with preserved ejection fraction (HFpEF). In fact, withdrawal of beta blockers in a small study of patients with HFpEF was associated with a reduction in natriuretic peptide (NT-proBNP) levels [112]. Similar benefit was demonstrated in a small pilot study using pacing at 80 bpm, suggesting that moderately higher heart rates might reduce LV filling pressures by shortening diastole in a stiff, non-compliant LV [113]. These findings were supported by the first randomized study in HFpEF patients with pacemakers, where personalized accelerated pacing improved quality of life and physical activity, reduced NT-proBNP, and lowered device-detected atrial fibrillation at 1 year compared to usual care [114]. Benefits appeared greater in patients with smaller LV volumes and higher LVEF [115]. Longer-term follow-up (~ 2 years) demonstrated changes in LV geometry, including reduced wall thickness, mild dilatation, and a small reduction in LVEF [116, 117]. Mechanistic studies further suggest that moderate increases in pacing rate lower left atrial pressure, whereas higher rates may be counterproductive due to AV interval prolongation [118]. Bachmann bundle pacing, intended to preserve more synchronous atrial activation, has shown favourable effects on biomarkers, diastolic parameters and exercise capacity [119]. Evidence supporting pacing-based treatment strategies in heart failure with preserved ejection fraction (HFpEF) thus remains preliminary, evaluating haemodynamic, biomarker, functional, and patient-reported endpoints. Potential risks include device infection, atrial arrhythmias, and uncertain long-term effects of sustained pacing on ventricular structure and function. Validation is awaited from the ongoing ELEVATE-HFpEF study (NCT06678841), randomizing ~ 700 HFpEF patients to personalized accelerated conduction system pacing—preferentially targeting the Bachmann bundle—versus no or minimal pacing, with clinical outcomes as primary endpoints.

### Implantable devices in heart failure monitoring

#### Monitoring of HF with Transvenous CIEDs

Despite major advancements in GDMT, the burden of worsening HF remains substantial, and contemporary data demonstrate that nearly half of worsening HF events occur outside the hospital–in emergency departments, observation units, and ambulatory clinics [120]. Trial evidence (STRONG-HF) and new outpatient diuretic options (subcutaneous furosemide, intranasal bumetanide) have accelerated a shift toward managing HF in the ambulatory setting, creating an urgent need for remote patient monitoring (RPM) strategies that can detect hemodynamic congestion before clinical deterioration [121]. Traditional surveillance using symptoms, vital signs, and body weight is well known to lack sensitivity for early decompensation, motivating sensor- and algorithm-based approaches that exploit existing CIEDs [122, 123].

**Fig. 3:**
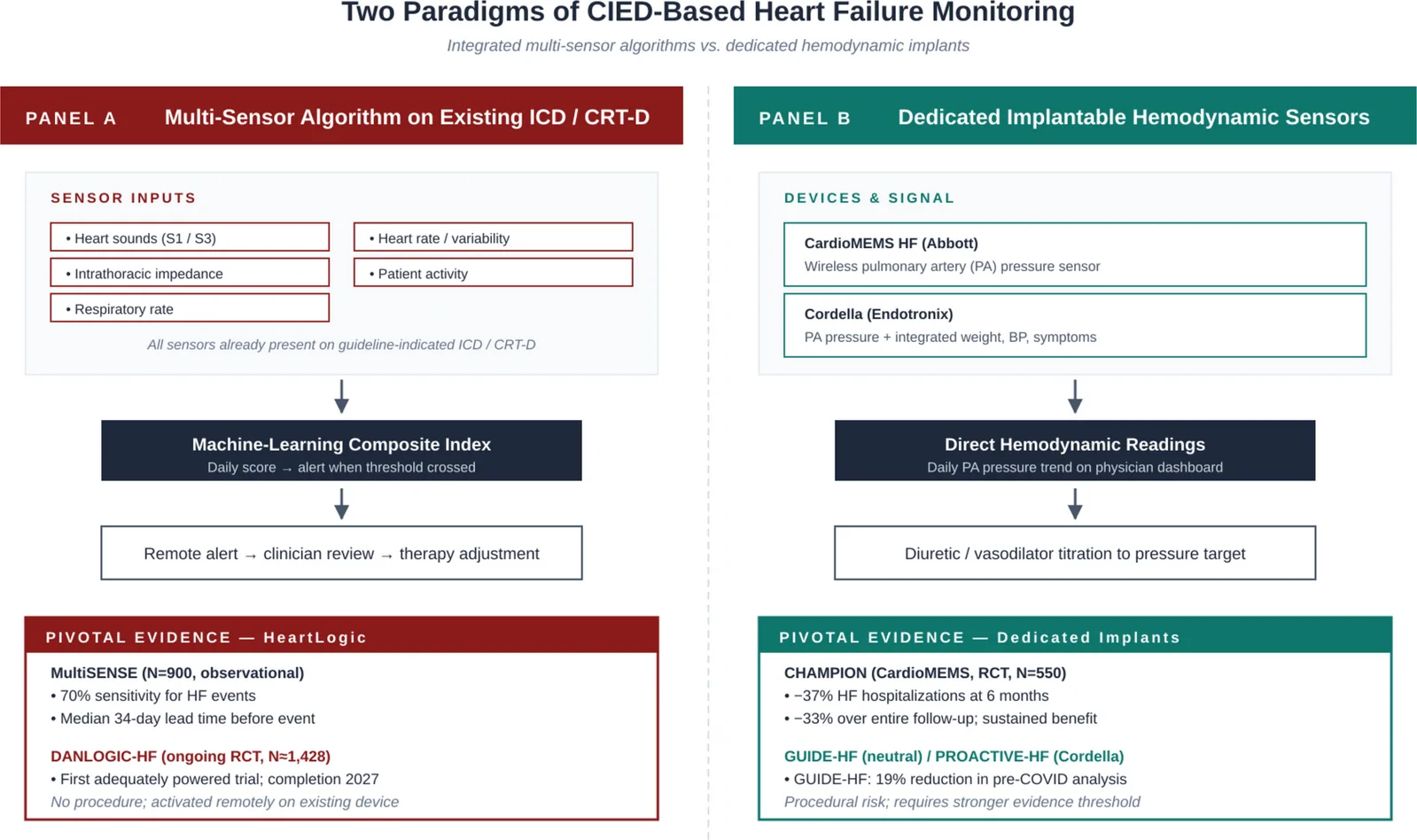
Two paradigms of CIED-based heart failure monitoring. **A** Multi-sensor algorithms (e.g., HeartLogic) leverage physiologic sensors already present on existing ICDs and CRT-Ds; pivotal evidence comes from the MultiSENSE study with the ongoing DANLOGIC-HF RCT [122–124]. **B** Dedicated hemodynamic implants (CardioMEMS, Cordella) directly measure pulmonary artery pressure; pivotal evidence comes from the CHAMPION trial, with mixed signals from GUIDE-HF and supportive single-arm data from PROACTIVE-HF [125–128]

Two parallel advances have made CIED-based monitoring increasingly attractive. First, modern ICDs and CRT pacemakers/defibrillators now carry multiple physiologic sensors–heart sounds (S1/S3), intrathoracic impedance, respiratory rate, heart rate variability, and patient activity—already in place for guideline-indicated therapy [122, 123]. Second, machine learning approaches have made it possible to fuse these channels into composite, personalized indices that outperform any single biomarker for the detection of impending decompensation [122]. Conceptually, two device classes have emerged: (1) sensors integrated into existing therapeutic CIEDs and (2) dedicated implants placed solely for monitoring. Each carries a distinct evidentiary threshold: dedicated implants incur procedural risk and therefore require stronger evidence of net clinical benefit, whereas integrated diagnostics piggyback on an already-indicated device and can be activated remotely (Fig. 3).

Early single-sensor strategies–most notably the OptiVol intrathoracic impedance index—produced disappointing results in randomized trials (DOT-HF, OptiLink HF), in large part because impedance changes lack specificity and generated frequent false alerts [129, 130]. The HeartLogic algorithm (Boston Scientific) represents the contemporary multi-sensor evolution of this approach [122, 123]. In the MultiSENSE study (N = 900 CRT-D recipients), HeartLogic detected 70% of HF events at a median of 34 days before the event, providing a clinically actionable lead time [122]. Real-world cohorts have since reported reductions in HF hospitalizations when alerts triggered structured clinical action [131–133]. Detection of alerts alone is not sufficient to reduce HF hospitalizations. To prevent worsening HF events, these physiologic sensor alerts must be reviewed promptly, interpreted in clinical context, and linked to an effective therapeutic response. A recent meta-analysis of 6 RCTs evaluating HF management guided by remote multiparameter monitoring found that this strategy reduced rates of heart failure hospitalization and all-cause mortality [134]. These benefits reflect not only improved detection of impending decompensation but also the ability to translate alerts into targeted HF management. The first adequately powered randomized trial of HeartLogic-guided care, DANLOGIC-HF (NCT06099158), is a pragmatic Danish registry-based RCT enrolling 1,428 patients with completion expected in 2027 [124].

Although uptake in routine practice has lagged the underlying technology, the convergence of high-fidelity device sensors, mature ML algorithms, and the policy imperative to keep patients at home is now driving meaningful integration of CIED-based monitoring into HF care pathways.

#### The evidence for implantable sensors to monitor HF

The most extensively studied dedicated implant is the CardioMEMS HF System (Abbott), a wireless pulmonary artery (PA) pressure sensor [125]. In the pivotal CHAMPION trial, 550 patients with NYHA class III HF and a prior HF hospitalization were randomized to PA pressure-guided management versus usual care [125]. PA-guided care reduced HF hospitalizations by 37% at 6 months and by 33% over the entire randomized follow-up, with benefit observed across the ejection fraction (EF) spectrum [125, 126]. The subsequent GUIDE-HF trial was neutral for the overall composite endpoint, although a pre-COVID analysis showed a 19% reduction driven by HF hospitalizations [127]. The Cordella Heart Failure System (Endotronix) is a second-generation PA sensor that also integrates daily vital signs, weight, and patient-reported symptoms; the PROACTIVE-HF trial reported a lower-than-expected HF hospitalization and mortality rate among device recipients, supporting FDA approval, though the single-arm performance-goal design limits causal inference [135]. Earlier-stage devices in development include left atrial pressure sensors (V-LAP) and inferior vena cava–based volume sensors.

Taken together, CIED-based monitoring has progressed from single-channel impedance alerts to validated multi-sensor algorithms and to dedicated hemodynamic implants with RCT evidence of benefit. Important gaps remain, procedural risk and cost for dedicated implants, alert fatigue and the absence of standardized response protocols for both categories, and limited representation of women, racial and ethnic minorities, and HFpEF patients in pivotal trials. The next generation of evidence–exemplified by DANLOGIC-HF and post-PROACTIVE-HF studies–should move beyond sensitivity and specificity to test, in adequately powered randomized trials with hard endpoints, whether acting on these signals with structured response protocols reduces HF hospitalization and mortality in real-world practice [131, 135]. CIED-based monitoring is most powerful when paired with rapid GDMT optimization and outpatient diuretic strategies–synergy, not substitution.

### Cardiac implantable electronic device procurement across Europe and real-world implementation

Globally, the conservative estimate is that at least 55 million people live with heart failure, and within Europe more contemporary assumption is exceeding 30 million patients [136, 137]. These numbers are increasing in last decades and pose a huge (unmet) challenge for modern medicine. Nonetheless, they also give stage for effective pharmacological and non-pharmacological therapy. Cardiac implantable electronic devices should be integrated into comprehensive HF management rather than viewed as stand-alone therapies. Device selection and timing should be coordinated with optimization of GDMT, particularly in ischemic cardiomyopathy, where β-blocker optimization or intolerance may influence arrhythmic risk, pacing requirements, and ICD or CRT decision-making. This integrated approach is especially relevant for CRT candidacy, ICD timing after myocardial infarction, reverse LV remodelling, and patients with bradycardia limiting optimization of medical therapy [138].

There is abundant evidence about suboptimal pharmacotherapy in HF, that spills over to CIED as evident from contemporary implantation statistics [139, 140]. As for all CIED, the heterogeneity persists also for HF specific devices, i.e. CRT, ICD and CSP, generally with ratio of 50 or more between countries with highest vs lowest rate of implantation. The decision about patient management always is with the attending clinician, and there are individual specifics in terms of perceptions, beliefs, attitudes and strategies that influence guideline interpretation and implementation [141]. At large, delay in pharmacological therapy delivery beyond 3–6 months and traditional drug sequencing strategies instead of simultaneous initiation appear to be most important challenges to tackle, and at least in part driven by objective reasons (e.g. hypotension, hyperkalaemia, and renal function worsening by patient worsening) or simply by patient non-adherence. As all these aspects prevent timely and evidence-based CIEDs use, the scientific community is developing the strategies to overcome therapeutic inertia through a comprehensive framework using up-to-date evidence and modern technologies [7]. These must be followed also once CIED is implanted. These pacing modalities generally should improve patient haemodynamic, abolish the bradycardia risk and therefore enable for therapy introduction and/or up-titration [142].

There may be many reasons for the current CIED implantation rates, including failure to implement guideline directed therapy, staff, reimbursement, and health policy in general. Many of these can be controlled with better and focused organisation yet some can truly be out of the community reach. A sobering message regarding the financial aspects can be extracted from a recent overview about public procurement practices in 22 European countries, that demonstrated heterogeneity with general lack of value-based principles, and limited integration of patient reported and clinical outcome into decision models [143]. On the other hand, it also identified procedures and structures that can instruct for optimization of value-based decision making across Europe.

## Conclusions

Cardiac implantable electronic devices have become an integral component of contemporary HF management, but their future lies not simply in technological innovation, but in delivering the right device to the right patient at the right time. Although advances in CSP, leadless technologies, and non-transvenous ICD systems have expanded therapeutic options, their widespread adoption should be guided by robust evidence demonstrating improvements in hard clinical outcomes beyond surrogate measures of electrical or echocardiographic response. Equally important is the integration of device therapy with GDMT and multidisciplinary heart failure care to maximize patient benefit. Finally, addressing persistent disparities in device utilization, improving timely referral, and ensuring equitable implementation of evidence-based therapies will be essential to fully realize the potential of CIEDs to improve outcomes in patients with HF.

## Data Availability

No datasets were generated or analysed during the current study.
